# Use of the Modified Ramberg-Osgood Material Model to Predict Dynamic Modulus Master Curves of Asphalt Mixtures

**DOI:** 10.3390/ma16020531

**Published:** 2023-01-05

**Authors:** Péter Primusz, Csaba Tóth

**Affiliations:** Department of Highway and Railway Engineering, Faculty of Civil Engineering, Budapest University of Technology and Economics, 1111 Budapest, Hungary

**Keywords:** master curve, shift factor, Ramberg-Osgood material model, asphalt mixture

## Abstract

Dynamic modulus master curves are usually constructed by using sigmoid functions, but the coefficients of these functions are not independent of each other. For this reason, it is not possible to clearly identify their physical mean. Another way of describing the dynamic modulus master curves is to choose the Ramberg-Osgood (RAMBO) material model, which is also well-suited for modelling the cyclic behaviour of soils. The Ramberg-Osgood model coefficients are completely independent of each other, so the evaluation of the fitted curve is simple and straightforward. This paper deals with the application of the Ramberg-Osgood material model compared to the usual techniques for constructing a master curve, determining the accuracy in describing the material behaviour of asphalt mixtures, and seeking any surplus information that cannot be derived by traditional techniques. Because the dynamic modulus and phase angle master curves are strictly related, in the present study, the asymmetric bell-shaped frequency curve of Toranzos was used to describe the phase angle for four types of asphalt mixtures (RmB, PmB, RA, and NB). The results show that the RAMBO model is a good alternative to the sigmoid function in describing the master curve of the dynamic modulus. We successfully used the Toranzos asymmetric bell-shaped frequency curve to describe the phase angle master curve. We also found a promising relationship between the independent RAMBO model parameters and the physical properties of the investigated binders, but this requires further research.

## 1. Introduction

It is a well-known fact that the behaviour of asphalt mixtures basically depends on the type of loading and the testing temperature. Consequently, asphalt mixtures can be regarded as viscoelastic materials for a significant part of their lifetime, as both viscous and elastic characteristics are simultaneously present, encumbering the analysis of the mixtures as well as the design of asphalt pavement structures.

According to the practice, one of the most important characteristics of a mixture is the dynamic modulus, that is, the absolute value of the complex modulus. The dynamic modulus is used in most mechanistic analyses of pavement response as well as the material input for the design of flexible pavement. Most frequently the dynamic modulus of a mixture is analysed under fixed conditions (i.e., at 15 °C and 10 Hz loading), since this parameter basically depends on the testing temperature and the loading time (it should be noted that, in many standards, the dynamic modulus of a mixture is analysed for different combinations of temperature and frequency, too).

The single stiffness value determined at a fixed temperature can be used for qualifying mixtures, however, the differences among various mixtures cannot be described thoroughly. The solution for this problem is provided by the master curve, based on the rheologic temperature-frequency similarity principle. The master curve is suitable for the detailed analysis of dynamic moduli and phase angles measured at various temperatures and frequencies; moreover, indirectly, the susceptibility to plastic deformation and cold cracking can be determined. The dynamic modulus is considered to be the most important factor of asphalt concrete that influences the field performance of asphalt pavement. The newly developed Mechanistic-Empirical Pavement Design Guide (MEPDG) also uses the dynamic modulus master curve to characterise the temperature and time-dependent behaviour of asphalt concrete.

In practice, master curves are usually constructed by applying a sigmoid function [1], although other function types and methods could be applied for this purpose as well [2]. A good example is the Christensen-Anderson (CA) and Christensen-Anderson-Marasteanu (CAM) models, whose morphological parameters are often used to investigate the effects of asphalt mixtures on the component properties [3,4,5]. The Witczak’s E* prediction equation also proves that the master curve parameters are related to the components of the mixtures (the volumetric properties of the mixture and viscosity of the bitumen) [6]. However, the coefficients of the Witczak’s sigmoid function are not independent of each other, which prevents researchers from understanding the physical meaning of the coefficients.

Another way to describe the master curves is to use the Ramberg-Osgood (RAMBO) material model [7], which is also well-suited to modelling the cyclic behaviour of soils. The coefficients of the Ramberg-Osgood model are independent of each other, so the evaluation of the curve fitted to the data is simple and straightforward.

This study intends to establish a unique identifier of an asphalt mixture by applying the RAMBO model, providing a useful basis for quality control systems both in asphalt production and construction. It is hoped that the new RAMBO model will be highly applicable to the determination of the dynamic modulus master curves of in-service asphalt layers using an FWD apparatus for mechanistic-empiric rehabilitation [8]. The research is still at an early stage, so this article summarises the results we have achieved so far.

## 2. Theoretical Background

### 2.1. Dynamic Characteristics of Asphalt Materials

In the design procedure, building materials of classic load-bearing structures are considered as having elastic behaviour. Elastic materials behave according to Hooke’s law, described by the well-known σ=E·ε formula. The proportional factor is the E elastic modulus, supposed to be constant. This material characteristic provides connection between stresses and strains. However, the behaviour of asphalt mixtures significantly varies from this supposed elastic behaviour, even at moderate range temperatures (5–15 °C), and especially at higher range (>40 °C) temperatures:

- Their elastic modulus is not constant but depends on the temperature and the loading time, and the frequency;
- In case of constant stress, the specific deformation increases over time (creeping);
- In case of constant specific deformation, the stress decreases over time (relaxation);
- In case of cyclic stress changes, hysteresis occurs with the dissipation of the mechanic energy.

These viscoelastic properties of the asphalt mixture come from the binder (the bitumen), characterised by its complex modulus and dynamic modulus. If, in the laboratory, an asphalt specimen gets a sinusoidal periodic excitation:

(1) $$\varepsilon \left(t\right)=\varepsilon_{0}\sin\left(\omega t\right)$$

**Figure 1:**
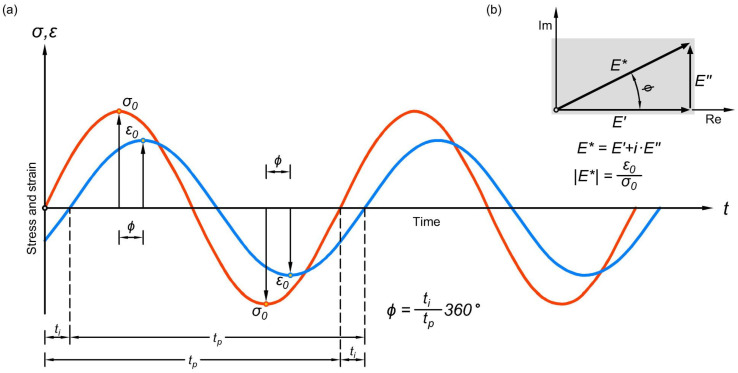
Response of the asphalt material to an excitation by a zero middle value clear sinusoidal pulse (**a**); the complex modulus and its components on the complex plane (**b**).

Then—because of the viscoelastic behaviour of the asphalt mixture—a phase shift occurs between the applied sinusoidal strain and the sinusoidal stress (Figure 1a):

(2) $$\sigma \left(t\right)=\sigma_{0}\sin\left(\omega t+\phi \right)$$

The ω circular speed is determined by the f frequency applied for the repeated loading (fatigue test):

(3) ω=2πf

Should the analysed material be elastic, the beginning and end points of the periods of the two sinus curves will overlap. The dynamic deformation modulus, called the complex modulus, is represented based on Hooke’s material law, but in a time-dependent way:

(4) $$E^{*}\left(t\right)=\frac{\sigma \left(t\right)}{\varepsilon \left(t\right)}$$

That is, to divide the σ and ε values connected to the t time moment.

The origin of the name of the complex modulus stems from the more advantageous representation of the $E^{*}$ value on the complex number plane. The complex modulus has a real $E^{\prime }$ elastic modulus part and an imaginary $E^{\prime \prime }$ viscous part. The real value of the complex modulus is the storage modulus because it stores the stress required for the relaxation of the deformation. The imaginary value of the complex modulus is the loss modulus, that is, the part of the stress usually dissipated as heat within the deformation process. The absolute value of the complex modulus (the dynamic modulus) can be calculated according to Figure 1b:

(5) $$\left|E^{*}\right|=\sqrt{{\left(E^{\prime }\right)}^{2}+{\left(E^{\prime \prime }\right)}^{2}}=\frac{\sigma_{0}}{\varepsilon_{0}}$$

In case of the calculation of the dynamic modulus, the $\sigma_{0}$ and $\varepsilon_{0}$ amplitudes are divided, regardless of their occurrence being at different moments but after each other, with a time difference represented by the ϕ phase delay.

The physical meaning of this mathematical result is that the value of the $E^{\prime }$ elastic modulus at moderate temperatures (20–25 °C) is rather close to the $\left|E^{*}\right|$ dynamic modulus, therefore the latter can be used as an elastic modulus for calculations. Based on the known dynamic modulus and phase angle, the storage and loss moduli can be calculated, too:

(6) $$E^{\prime }=\left|E^{*}\right|\cos\phi$$

(7) $$E^{\prime \prime }=\left|E^{*}\right|\sin\phi$$

since:

(8) $$\tan\phi =\frac{E^{\prime \prime }}{E^{\prime }}$$

A final remark: the ϕ phase angle of elastic materials is 0°, while the phase angle of viscous materials is 90°. The phase angle of viscoelastic materials varies between 0° and 90°.

### 2.2. Time Temperature Equivalence Principle

The time temperature equivalence principle states that the relaxation time parameters of simple thermo-rheologic materials vary at the same rate by the effect of temperature change. Consequently, quantities depending on the loading time, such as the $E^{*}$ complex modulus of asphalt mixtures, can be shifted along the time axis according to the temperature changes, therefore material characteristics measured at various temperatures can be put together in a diagram called the master curve.

The time-temperature equivalence principle makes possible the construction of a master curve based on data collected at various temperatures and loading frequencies, using a reference temperature or frequency for horizontal shifting, harmonizing different isotherms.

The superposition mathematically can be realised, introducing a so-called reduced frequency. The $\alpha_{T}$ shift factor determines the required shifting along the horizontal axis at a given temperature. The real f frequency shall be multiplied by this shift factor, in order to get the reduced frequency value of the master curve:

(9) $$f_{r}=\alpha_{T}\cdot f$$

(10) $$\log\left(f_{r}\right)=\log\left(\alpha_{T}\right)+\log\left(f\right)$$

At the reference temperature, the shift factor gets the value of $\alpha_{T}=1$.

The master curve for a given reference temperature can be established by shifting the isotherms related to other temperatures, parallel to strictly only the loading time or frequency axis. In the practice, the Arrhenius equation is often used for the determination of the value of the shift factor [9]:

(11) $$\log\left(\alpha_{T}\right)=\underset{C_{A}}{\underbrace{\frac{0.4347\cdot ∆E_{a}}{R}}}\left(\frac{1}{T}-\frac{1}{T_{0}}\right)=C_{A}\cdot \left(\frac{1}{T}-\frac{1}{T_{0}}\right)$$

where

$\alpha_{T}$ = shift factor;

T = testing temperature (K);

$T_{0}$ = reference temperature (K);

$∆E_{a}$ = activation energy (J mol^−1^);

R = universal gas constant (8.314 J mol^−1^ K^−1^);

$C_{A}$ = constant (K).

In order to describe the behaviour of the material applying the Arrhenius equation, only the value of the $C_{A}$ constant shall be determined.

In rheology, besides the Arrhenius equation, another frequently used formula is the Williams-Landel-Ferry (WLF) equation for the determination of the value of the shift factor [10]. Both classic shift factor determination methods can be applied for analysis of bitumen and asphalt mixtures, although these methods have been prepared mainly for polymers, therefore their application nowadays is somewhat reduced [11].

In case of a large amount of measured data, it is possible, instead of classic shift factor applications, to handle the shift factor as an independent variable, in order to determine the best-fitted function parameters, according to data available, applying some kind of an iteration technique.

For optimisation, the international literature recommends characteristically a second-order function for the approximation of the shift factor; for example:

(12) $$log\left(\alpha_{T}\right)=a\cdot {\left(T-T_{0}\right)}^{2}+b\cdot \left(T-T_{0}\right)$$

where

$\alpha_{T}$ = shift factor;

T = testing temperature;

$T_{0}$ = reference temperature;

a,b = parameters.

The quadratic polynomial Equation (12) provides a good fitting for shift factors at a wide range of temperatures.

The log-linear model is one of the most popular methods for shifting by temperatures in case of asphalt mixtures. According to Christensen and Anderson [12], slightly below 0 °C, for many binders, the quantity $log\left(\alpha_{T}\right)$ varies linearly by the temperature, therefore this formula has been recommended for asphalt mixtures at low and medium temperatures. The log-linear equation for the calculation of the shift factor is:

(13) $$\log\left(\alpha_{T}\right)=C_{1}\cdot \left(T-T_{0}\right)$$

where $C_{1}$ is the gradient of the linear line between $\log\left(\alpha_{T}\right)$ and the temperature. Further detailed information related to shift factors can be found in the excellent work of Rowe and Sharrock [13].

### 2.3. The Master Curve of the Dynamic Modulus

The master curve of the dynamic modulus of an asphalt mixture can be well-described using a non-linear S-shaped sigmoid function. The applicability of the sigmoid function has been verified by the physical observation of the behaviour of the mixture. Its general formula is:

(14) $$f\left(x\right)=\frac{1}{1+e^{-x}}$$

Based on measurement results, the dynamic moduli, the master curve of the mixture, can be constructed. In the case of asphalt mixtures, the formula of the master curve depends on the frequency [1]:

(15) $$\log\left|E^{*}\right|=\delta +\frac{\alpha }{{1+e}^{\beta +\gamma \cdot \log\left(f_{r}\right)}}$$

where

$\left|E^{*}\right|$ = dynamic modulus of the asphalt mixture (MPa);

$f_{r}$ = reduced frequency (Hz);

δ = minimum of the dynamic modulus (MPa);

δ+α = maximum of the dynamic modulus (MPa);

β,γ = parameters describing the shape of the function.

A significant disadvantage of the model is that it uses only the absolute value of the $E^{*}$ complex modulus of the mixture, the dynamic modulus, and, consequently, the viscoelastic effect indicated by the phase angle cannot be analysed in the behaviour of the mixture. This fact leads to the conclusion that the sigmoid model is not in consonance with real test results. The presented (15) sigmoid model has a generalised version (GSM) that is well-fitted into the non-symmetrical data points, too, as presented in the work of Rowe, Baumgardner, and Sharrock [14].

In parallel with empirical sigmoid functions, however, there are also physically based rheological models based on fractional differential equations, which are essentially spring-dashpot-springpot combinations [15]. The advantage of these approaches is that a closed physical-mathematical concept is obtained, based on time-differential equations, which can be evaluated for arbitrary loads (creep, cyclic loads). The master curves are then directly derivable from the concept [16].

### 2.4. The Master Curve of the Phase Angle

The master curve of the phase angle is usually constructed similarly to the master curve of the dynamic modulus, based on experimental data. However, this part of the modelling gets less interest, compared to the issues of the dynamic modulus. The causes of this fact are, on one hand, that describing the shape of the ϕ master curve is a much more difficult task, and on the other hand, that the knowledge of the phase angle is not necessary for the mechanistic-empirical road pavement design procedure based on the linear elasticity [17].

Despite difficulties, there is a need to study the phase angle as well, because, in the process of fitting the master curve of the dynamic modulus to data, not considering the phase angle is not permissible, since only considering the phase angle provides a good basis for an assumable physical meaning of the model.

One practical trend for constructing the master curve of the phase angle is based on bell-shaped functions. It is possible to apply the enhanced Gaussian curve [18], the Beta distribution [19], and the Lorenz curve (Cauchy distribution) with 3 or 5 parameters [20,21]:

(16) $$\phi \left(f_{r}\right)=\frac{a\cdot b^{2}}{{\left[\log\left(f_{r}\right)-c\right]}^{2}+b^{2}}$$

where

$f_{r}$ = reduced frequency (Hz);

a = peak value;

b = growth rate;

c = critical point.

Moreover, an empirical formula of Bahia et al. [22] is often applied in research studies to model the phase angle [23].

Another possibility is the use of Kramers–Kronig relations, which are mathematical formulas for connecting the real and imaginary parts of complex analytic functions. The practical significance is given by the possibility of determining the real part of the response of physical systems based on the knowledge of the complex part (and to determine the complex part based on the knowledge of the real part, respectively). According to the approximate formula of Booij and Thoone [24], the shape of the phase angle function can be deduced from the connection between the dynamic modulus and the loading frequency. Later, the original formula has been amended by Yang and You [2] to include a c coefficient in order to get a potentially better estimation:

(17) $$\phi \left(\omega \right)\approx c\frac{\pi }{2}\frac{d\log\left(\left|E_{\omega }^{*}\right|\right)}{d\log\left(\omega \right)}$$

where c is a positive coefficient and $\omega =2{\pi f}_{r}$. The formula (16) makes possible to deduce the mathematical model of phase angle from the (14) sigmoid model of dynamic modulus [25].

## 3. Material and Method

### 3.1. Introduction of the Analysed Material

In this study, basically four types of asphalt mixtures have been analysed. The rubber-modified (RmB), the polymer-modified (PmB), and the reclaimed asphalt-contained (RA) mixtures have been compared to a reference mixture with conventional binder (NB). These are the analysed mixtures:

- AC22 binder NB 50/70
- AC22 binder RA 70/100
- AC22 binder PmB 25/55–65
- AC22 binder RmB 45/80-55

Three specimens from the four analysed asphalt mixture types—the NB 50/70, the PmB 25/55-65, and the RmB 45/80-55—have been mixed using average values of the upper and lower limit values of the particle distribution curve of the AC22 mix. The specimens of the reclaimed asphalt contained RA 70/100 mixture have been mixed using only the upper and lower limit values of the particle distribution curve of the AC22 mix. This way these mixtures aimed to analyse the effect of the mineral skeleton on the master curves. The bitumen content was in three cases 4.5 mass % (m%). In case of the RA 70/100 mixture, the bitumen content was 1.2 m% RA and 3.3 m% NB 70/100 added bitumen. The most important physical characteristics of the binders are summarised in Table 1.

In the polymer modified binder (PmB) a spatial mesh structure between the polymer molecule and the bitumen increases the resistance to deformation at high temperatures, resulting in a lower penetration and higher softening point [26]. The rubber bitumen (RmB) is a binder for road construction made from the crumb rubber of used tyres and possibly other additives [27]. The behaviour of the RmB binder is similar to that of the NB, showing more favourable properties in cold temperature ranges [28].

### 3.2. Introduction of the Testing Procedure

**Figure 2:**
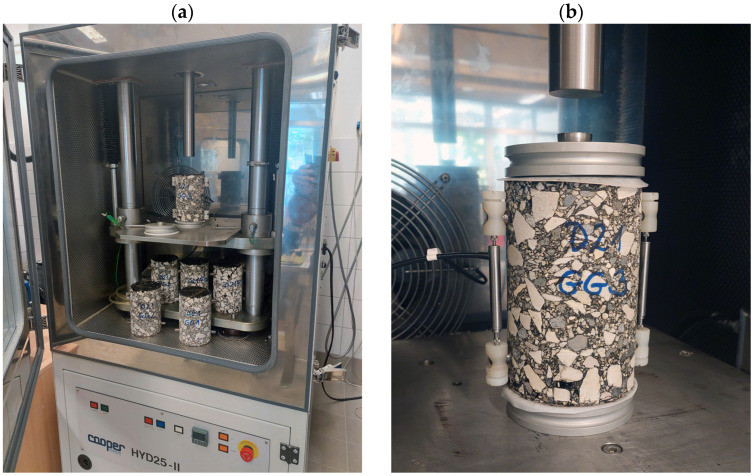
Complex modulus analysis in the study: the dynamic analyser device (HYD25-II) (**a**); the specimen prepared for testing the complex modulus (**b**).

The complex moduli and phase angles of the four asphalt mixtures have been calculated from data acquired using the Simple Performance Tester (SPT) or the Asphalt Mix Performance Tester (AMPT) devices manufactured by Cooper Co., Ripley, UK (Figure 2a). A minimum of two specimens have been made of each mixture for testing. The reason for the low sample number is that we did not have more asphalt components available to prepare further mixtures. The specimens have been tested at four temperatures and at six different frequencies.

- Temperatures (T): 0 °C, 10 °C, 20 °C, 30 °C
- Frequencies (f): 0.1 Hz, 0.5 Hz, 1 Hz, 5 Hz, 10 Hz, 25 Hz

A 60 s resting time was left between each frequency, providing a certain regenerative period for the specimens, before applying a new loading at a lower frequency. The testing was performed using controlled stress, therefore the highest specific deformation remained below 200 microstrains. This way, the reaction of the material at the testing temperature became linear. The deformations in the axis direction were measured using two linear variable differential transformers (LVDT) manufactured by Cooper Co., Ripley, UK, placed vertically on the reverse sides at the diameter of the specimen. In order to eliminate the development of shear stresses at the end of specimens within the tests, a rubber membrane lubricated with vacuum grease was fastened to the top and the bottom of each specimen (Figure 2b).

Data have been acquired for all 24 combinations of temperatures and frequencies, and based on these data, the representative master curves have been constructed for each mixture.

### 3.3. The RAMBO Model of the Dynamic Modulus

Master curves of asphalt mixtures are described traditionally using sigmoid functions (SM or GSM), although other function types could be applied for this purpose as well. One such possibility is the Ramberg-Osgood elastic-plastic material law, originally and often used for modelling the cyclic behaviour of soils [29]. The Ramberg-Osgood model describes the non-linear connection between the stress and strain of the material and its yield limit value:

(18) $$\varepsilon =\frac{\sigma }{E}+C{\left(\frac{\sigma }{E}\right)}^{R}$$

where ε is the specific strain, σ is stress, E is the Young-modulus, and C and R are constants depending on the analysed material. The general mathematical formula of the Ramberg-Osgood (RAMBO) model is:

(19) $$x=y+Cy^{R}$$

The primal paper of Kweon recommends the application of the above-mentioned function for describing the master curve of asphalt mixtures, with replacements of $x=f_{r}$ and $y=E_{N}f_{r}$ in the following form [7]:

(20) $$f_{r}=E_{N}f_{r}+C{\left(E_{N}f_{r}\right)}^{R}$$

where $E_{N}$ is the normalised dynamic asphalt modulus:

(21) $$E_{N}=\frac{\left|E^{*}\right|-{\left|E^{*}\right|}_{min}}{{\left|E^{*}\right|}_{max}-{\left|E^{*}\right|}_{min}}$$

$f_{r}$ is the reduced frequency, and C and R are model constants.

The fitting of the RAMBO model to the measured data is possible using optimisation methods, similarly to the case of sigmoid functions. The result of the fitting provides material parameters ${\left|E^{*}\right|}_{min}$, ${\left|E^{*}\right|}_{max}$, C, and R.

Kweon [7] has proven that the parameters in the RAMBO model independently influence the master curve (Table 2). The R parameter influences the shape (curvature) of the master curve, while the C parameter provides shifting along the horizontal axis, similarly to the shift factor of temperature-time. This latter statement was not analysed in detail by the author. The independence of the parameters in the RAMBO model makes possible the numerical characterisation of the entire morphology of the master curve, that furthermore provides a possibility for a quick comparison of some mixture types. Since, from Equation (20), the $E_{N}$ cannot be solved in case of a general R, a solution exists only for the $f_{r}$, henceforward, the following formula is suitable for the analysis:

(22) $$f_{r}={\left(\frac{C_{0}\cdot E_{N}}{1-E_{N}}\right)}^{\frac{1}{1-R}}$$

where $f_{r}$ is the reduced frequency, $C_{0}$ is the shift parameter at the $T_{0}$ reference temperature, and R<1 is the shape parameter for the curvature of the master curve. Using Equation (9) and substituting it into Equation (22):

(23) $$f=\frac{1}{\alpha_{T}}{\left(\frac{C_{0}\cdot E_{N}}{1-E_{N}}\right)}^{\frac{1}{1-R}}$$

The RAMBO model provides the master curve of the asphalt mixture, depending on the f frequency. For the $\alpha_{T}$ shift factor, the log-linear model has been chosen, based on our former study, proving that the change of the $C_{0}$ parameter depending on the temperature can be well-described by an exponential function [30]:

(24) $$\ln\left(\alpha_{T}\right)=C_{1}\left(T-T_{0}\right)$$

where $\alpha_{T}$ is the shift factor, T is the temperature, $T_{0}$ is the reference temperature (20 °C), and $C_{1}$ is the constant determined by analysing the experimental data. Substituting the log-linear model into Equation (23), after necessary alterations, the final model Equation is:

(25) $$f={\left[C_{0}exp\left\{B∆T\right\}\frac{E_{N}}{1-E_{N}}\right]}^{\frac{1}{1-R}}$$

where $B=C_{1}\left(R-1\right)$ and $∆T=T-T_{0}$ substitutions have been applied. The parameters in the (25) RAMBO model can be determined by minimising the square error of the frequencies from the experiment compared to the model estimation:

(26) $${SSE}_{f}=\sum_{i=1}^{N}{\left(\frac{f_{m,i}-f_{p,i}}{f_{m,i}}\right)}^{2}$$

where $f_{m,i}$ is frequency of the ith experiment, $f_{p,i}$ is frequency (ith) estimated by the RAMBO model, and N is number of test specimens (dynamic moduli). The error function is suitable for determining the $f_{p,i}$ frequencies best fitted to the measured ${\left|E^{*}\right|}_{m,i}$ dynamic moduli. Since a right assumption is that, within the testing procedure, the adjusted frequencies have only a minor error, the correction for the dynamic modulus values shall provide ${SSE}_{f}=0$. For this purpose, the $\vec{CF}=\left[{CF}_{1}{CF}_{2}...{CF}_{N}\right]$ correction factors have been introduced, for correcting the measured ${\left|E^{*}\right|}_{m,i}$ dynamic moduli, in order to minimise the square error of the measured and corrected dynamic asphalt moduli:

(27) $${SSE}_{N}=\sum_{i=1}^{N}{\left(\frac{{\left|E^{*}\right|}_{m,i}-{CF}_{i}{\left|E^{*}\right|}_{m,i}}{{\left|E^{*}\right|}_{m,i}}\right)}^{2}$$

Based on this formula, it is obvious that the ${\left|E^{*}\right|}_{p,i}$ dynamic modulus, estimated by the RAMBO model, is equalled by the corrected one:

(28) $${{\left|E^{*}\right|}_{p,i}=CF}_{i}{\left|E^{*}\right|}_{m,i}$$

For the determination of the parameters of the RAMBO model, error functions (26) and (27) shall be minimised simultaneously:

(29) $$e_{f}=\underset{}{\min}\left({SSE}_{f}+{SSE}_{N}\right)$$

The results of the model fitting procedure are the parameters R, $C_{0}$, B, ${\left|E^{*}\right|}_{min}$, and ${\left|E^{*}\right|}_{max}$ of the RAMBO model, as well as the $\vec{CF}$ correction factor.

### 3.4. The Toranzos Model of the Phase Angle

In this study, the asymmetric bell-shaped frequency curve, developed by Toranzos, has been applied to describe the ϕ phase angle. The general mathematical formula of the model [31] is:

(30) $$y=k\cdot exp\left\{-0.5a^{2}{\left(x-b\right)}^{2}\right\}x^{c}$$

Since the exponent of the $x^{c}$ function can be a fraction, the formula is valid only in case of x>0. However, the ϕ phase angle is bell-shaped only on a logarithmic frequency scale, therefore the $f_{0}$ shift parameter has been introduced, and the initial value of it can be calculated as:

(31) $$\log f_{0}=min\left(\log\vec{f_{r}}\right)-1$$

where $\vec{f_{r}}=\left[f_{1}f_{2}...f_{N}\right]$. Applying the substitutions $x=\log\left(f_{r}/f_{0}\right)$ and y=ϕ, the model of the ϕ phase angle becomes:

(32) $$\phi =k\cdot exp\left\{-0.5a^{2}{\left(\log\left(\frac{f_{r}}{f_{0}}\right)-b\right)}^{2}\right\}{\log\left(\frac{f_{r}}{f_{0}}\right)}^{c}$$

The parameters in the Toranzos model (32) can be determined while minimising the square error of the experimental ϕ phase angles compared to those estimated by the model:

(33) $${SSE}_{\phi }=\sum_{i=1}^{N}{\left(\frac{\phi_{m,i}-\phi_{p,i}}{\phi_{m,i}}\right)}^{2}$$

where $\phi_{m,i}$ is the phase angle measured in the ith experiment, $\phi_{p,i}$ is the phase angle (ith) estimated by the Toranzos model, and N is the number of test specimens. The optimisation task has been expanded to include the $f_{0}$ shift parameter besides the a, b, c, and k parameters, considering a constraint of $logf_{0}\geq min\left(\log\vec{f_{r}}\right)-1$, providing better model fitting for the data in some cases.

### 3.5. The Model Fitting Procedure

Since the master curves of the $\left|E^{*}\left(f_{r}\right)\right|$ dynamic modulus and the $\phi \left(f_{r}\right)$ phase angle are in strict correlation, the RAMBO and the Toranzos models, presented in the current study, shall be fitted into experimental data simultaneously, minimising all square errors concerned:

(34) $$e_{f}=min\left({SSE}_{f}+{SSE}_{N}+{SSE}_{\phi }\right)$$

For the optimisation, the Microsoft Excel Solver tool has been used. Out of the model parameters, for the initial values of ${\left|E^{*}\right|}_{min}$ and ${\left|E^{*}\right|}_{max}$, it is purposeful to choose the minimum and maximum dynamic moduli of experimental data, assuming there is no other rational constraint.

### 3.6. Assessment of the Quality of the Fit

In case of all regression models, the standard error of the estimation of y relevant to x ($S_{e}$), the standard deviation ($S_{y}$), and the adjusted coefficient of determination ($R_{*}^{2}$) have been calculated. The standard error of estimation has been calculated as follows:

(35) $$S_{e}=\sqrt{\frac{\sum {\left(y-\hat{y}\right)}^{2}}{n-k}}$$

where n is the sample size, k is the number of independent variables, y is the measured value, and $\hat{y}$ is the predicted value. The standard deviation of the sample is:

(36) $$S_{y}=\sqrt{\frac{\sum {\left(y-\bar{y}\right)}^{2}}{n-1}}$$

where $\bar{y}$ is the average of measured values, and the other variables are as beforehand. The $S_{e}/S_{y}$ standard error ratio and the adjusted coefficient of determination ($R_{*}^{2}$) have been used for the assessment of the quality of fit between the measured and predicted values [32]. The adjusted $R_{*}^{2}$ is a modification of the $R^{2}$ considering the number of observations and the number of explanatory variables:

(37) $$R_{*}^{2}=1-\frac{n-k}{n-1}{\cdot \left(\frac{S_{e}}{S_{y}}\right)}^{2}$$

The smaller the $S_{e}/S_{y}$ is and the higher the $R_{*}^{2}$ is, the better the fit of the predicted and measured data is. Based on former research, the model fit is excellent if the $S_{e}/S_{y}$ ratio is less than 0.35 and the value of the $R_{*}^{2}$ is more than 0.9, according to Table 3 [33,34].

## 4. Evaluation of Results

**Figure 3:**
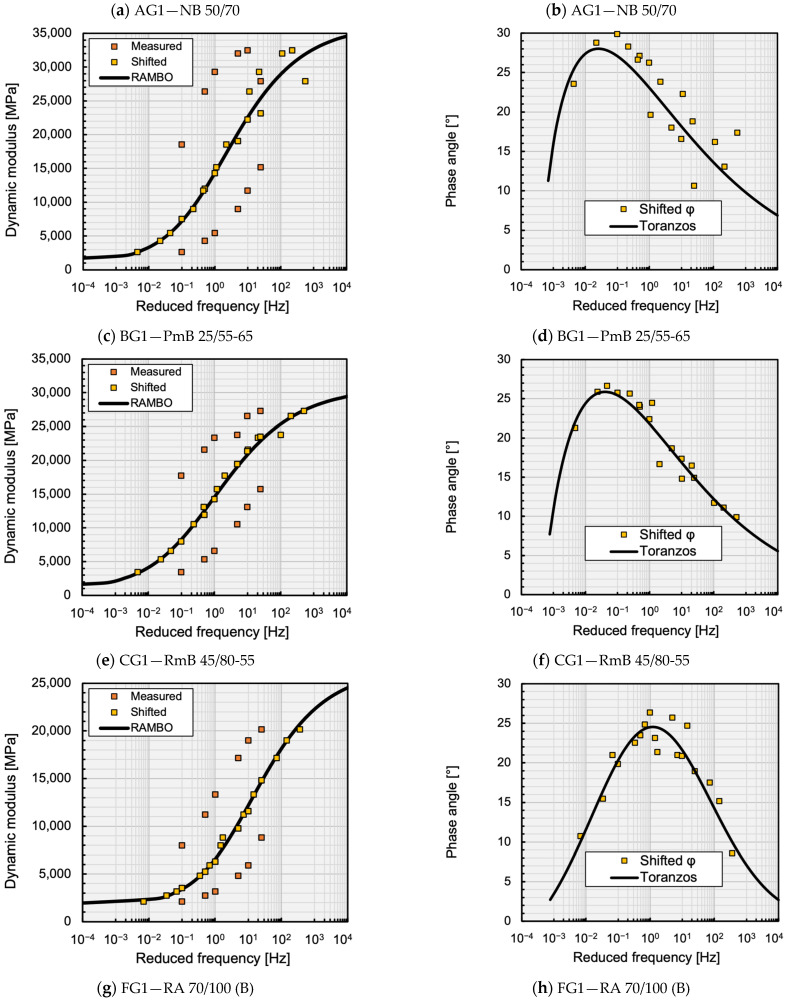

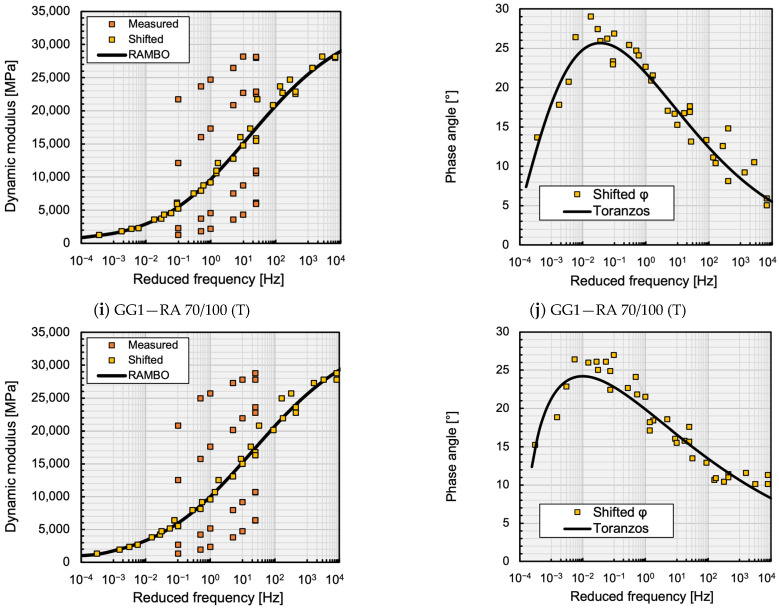
Dynamic modulus (**a**,**c**,**e**,**g**,**i**) and phase angle (**b**,**d**,**f**,**h**,**j**) master curves for T_0_ = 20 °C.

Figure 3 present graphically the fitting of the master curves of the dynamic modulus and the phase angle, plotted as examples of the mixtures analysed. The average parameters of the RAMBO and Toranzos master curves are shown in Table 4 for each mixture. Table 5 summarises the statistical evaluation of the quality of the model fits.

In general, it can be stated that the excellent fit criterion of the master curves of the $\left|E^{*}\right|$ dynamic moduli predicted by the RAMBO model has been fulfilled for all asphalt mixtures. The RAMBO model has shown the best fit in the case of the rubber-modified bitumen mixture CG1, finding the lowest $S_{e}/S_{y}=0.0682$ and the highest $R_{*}^{2}=0.9939$ values. The performance of the Toranzos phase angle model has been slightly weaker, but the fitting is still considered as good, according to Table 3. This model has shown the lowest $S_{e}/S_{y}=0.2414$ and the highest $R_{*}^{2}=0.9238$ values in the case of the polymer modified bitumen mixture BG1. The worst fitting has been found in the case of the conventional bitumen mixture AG1, showing a “Good” $S_{e}/S_{y}=0.5024$ value and a “Fair” $R_{*}^{2}=0.6699$ value.

**Figure 4:**
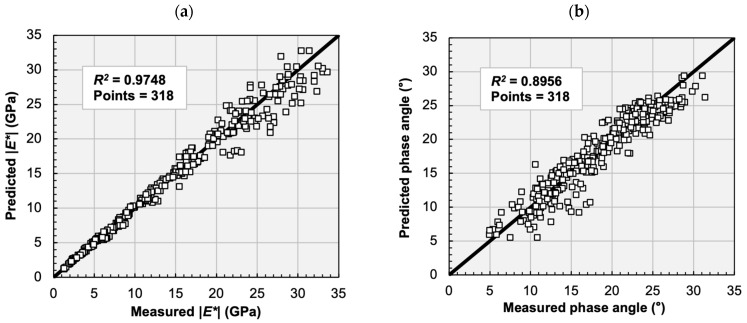
Comparison of predicted and measured values of dynamic moduli (**a**) and phase angles (**b**) in case of 5 asphalt mixture types (318 data points).

Figure 4a shows the comparison of the $\left|E^{*}\right|$ dynamic modulus values of the analysed asphalt mixtures by plotting the laboratory measurements and predictions of the RAMBO model, based on 318 data points. On the whole, the RAMBO model has provided a good prediction for the $\left|E^{*}\right|$ dynamic modulus values, since a high correlation (*R*^2^ = 0.9748) has been observed between the measured and predicted data. Nevertheless, compared to former studies, the prediction ability of the RAMBO model is slightly weaker (*R*^2^ < 0.99) than that of the sigmoid equation [19,35]. The main reason for this fact is that, in the present study, the phase angle data have been considered in the determination of the parameters of the RAMBO model, which decreased the prediction ability of the model. Secondly, the lower accuracy can be explained by the higher standard deviation of the data measured at low temperatures.

Figure 4b shows the comparison of the ϕ phase angle values of the analysed asphalt mixtures by plotting the laboratory measurements and predictions of the Toranzos model, again based on 318 data points (the same data points as in Figure 4a). The measured phase angle varied between 5° and about 30°. The phase angle values predicted by the Toranzos model have shown a strong linear correlation with the measured values, although, in general, the predicted phase angle values have been slightly lower, compared to the measured values. These results indicate that the Toranzos model can predict the phase angle with good accuracy. Nevertheless, the overall accuracy of the prediction of the phase angle is lower (*R*^2^ = 0.8956) than in the case of the prediction of the dynamic modulus, which can be explained by the lower accuracy of the phase angle data. This fact is verified by former studies, stating that, sometimes, it is impossible to construct a smooth master curve for the phase angle data [2,36].

The arbitrarily chosen log-linear $\alpha_{T}$ shift factor for the construction of the master curves proved to be suitable in the cases of all the asphalt mixtures analysed. The relationship between the logarithm of the shift factor ($\log\alpha_{T}$) and the temperature (T) remained linear within the analysed temperature range in the cases of all the asphalt mixtures analysed.

**Figure 5:**
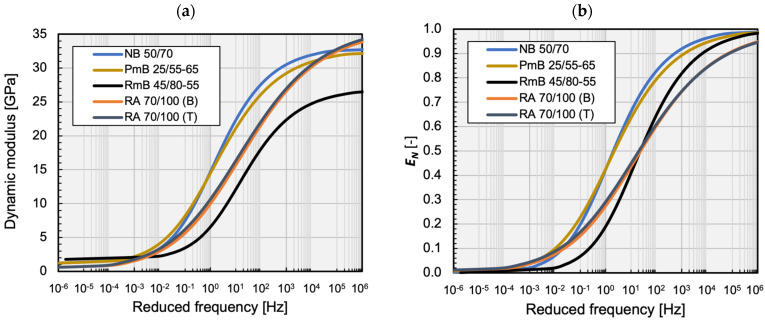
RAMBO master curves of the dynamic modulus (**a**) and the normalised dynamic modulus (**b**) depending on the reduced frequency.

**Figure 6:**
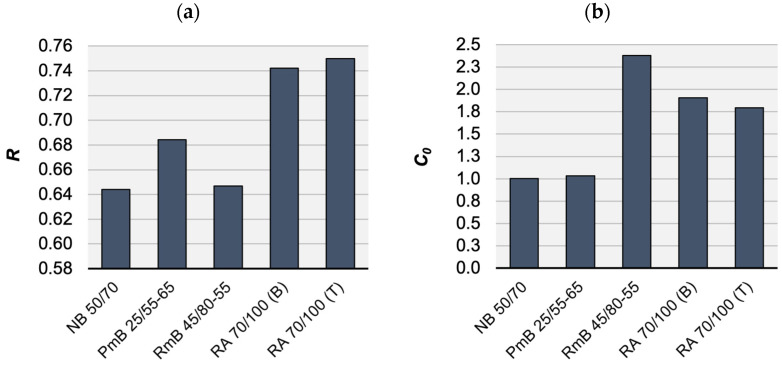
RAMBO model parameters of analysed mixtures: (**a**) average R curvature and (**b**) average $C_{0}$ shift by mixture type.

Figure 5a shows the average RAMBO master curves of the analysed mixtures. In order to better study the parameters of the RAMBO model, the normalised dynamic modulus has been plotted, depending on the reduced frequency, as well (Figure 5b). It is easily visible that the curves of the mixtures containing conventional and polymer-modified binders run close to each other, as their $C_{0}$ parameter is almost equal (Figure 6b). The only difference is caused by the R curvature parameter (Figure 6a). Another R value like the curvature of the conventional binder mixture has been found in the case of the rubber-modified mixture, but its curve has been shifted into higher frequencies because of its higher $C_{0}$ parameter. This shift may predict more favourable fatigue characteristics in the cold temperature range.

Specimens mixed with reclaimed asphalt for the bottom RA 70/100 (B) and top RA 70/100 (T) limit values of the particle distribution curve of the AC22 mix have shown very similar R and $C_{0}$ parameters. The influence of the stone skeleton has appeared only in higher ${\left|E^{*}\right|}_{max}$ and R values in the case of the specimen RA 70/100 (T) mixed for the top limit values. No significant difference in particle distribution has been found between the two RA 70/100 mixtures.

The RA 70/100 mixtures have the lower ${\left|E^{*}\right|}_{min}$ value but the highest ${\left|E^{*}\right|}_{max}$ and R curvature. This fact indicates that, in the cases of the mixtures containing reclaimed asphalt, a higher stiffness can be expected at higher temperatures [37]. In summary, it can be stated that the R parameter can relate to the stiffness of the asphalt mixture, including the influence of the stone skeleton and the binder.

**Figure 7:**
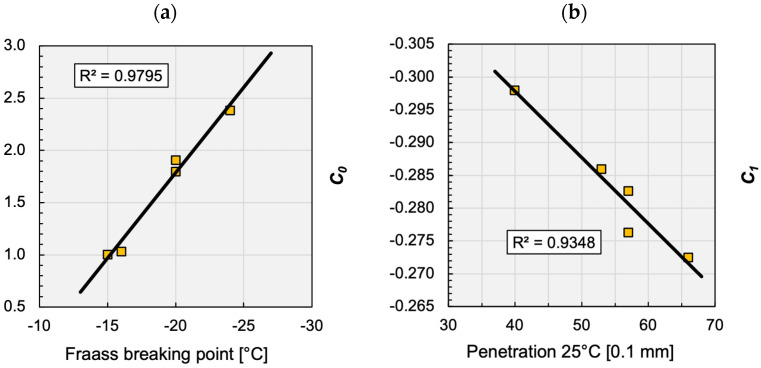
Assumed relationship between RAMBO model parameters: (**a**) Fraass breaking point vs. *C*_0_ and (**b**) penetration vs. *C*_1_.

A possible connection of the $C_{0}$ and $C_{1}$ parameters with the rheological parameters of the binder is shown in Figure 7a,b. The high correlation in this case does not prove the existence of the relationship since these figures have been plotted from very few data. By all means, as the $C_{0}$ and $C_{1}$ parameters mainly depend on the T temperature, the existence of the relationship through the viscosity of the binder can be rightfully assumed. In the future, after analysing big databases of asphalt mixtures, this hypothesis can be accepted or rejected.

## 5. Conclusions

The single dynamic modulus value determined at a prescribed temperature can be used for the qualifying mixtures, however, differences among the various mixtures cannot be described thoroughly. Considering that the time-temperature equivalence provides a possibility for studying special time and frequency domains that cannot easily be tested experimentally, an important task is to develop and refine suitable mathematical tools. Applying these tools in the construction of the master curve of asphalt mixtures, a comparison will be possible among tests performed at various frequencies and temperatures; moreover, the physical behaviour of the asphalt mixtures on the full temperature scale can be observed and registered along with the stiffness testing.

In the present study, a possible method for the construction of the master curve of the asphalt mixture has been prepared: applying the RAMBO model besides the sigmoid function. According to the analysis performed, the RAMBO model can substitute well for the sigmoid function in the description of the master curve of the dynamic modulus. Moreover, the independence of its parameters makes possible the numerical characterisation of the entire morphology of the master curve, which furthermore provides a good basis for quality control systems in asphalt production and pavement construction. In the future, an important research topic would be the analysis of the relationship between parameters of the RAMBO model and physical characteristics of the binder. The asymmetric bell-shaped frequency curve, recommended by Toranzos, has been successfully applied in the description of the master curve of the phase angle. Using this approach, the master curve of the phase angle could be constructed rather well, considering the accuracy of the laboratory-measured phase angle data. Detailed analysis of the parameters of the Toranzos model has not been performed in the present study, since the emphasis has been put on the RAMBO model, but some research work will be started into this direction soon as well.

## Figures and Tables

**Table 1:** Physical characteristics of the binders of asphalt mixtures.

Physical Characteristic	NB 50/70	RA 70/100	RmB 45/80-55	PmB 25/55-65
Penetration 25 °C (0.1 mm)	53	57	66	40
Softening point (°C)	52	54	58	79
Fraass breaking point (°C)	−15	−20	−24	−16

**Table 2:** Effect of parameters on the master curve by Kweon [7].

Model Type	Parameter	Master Curve			
		Modulus		Shape	
		Min.	Max.	Curvature	Shift
Sigmoid model	δ	●	●	×	×
	α	×	●	×	●
	β	×	×	●	×
	γ	×	×	×	●
RAMBO model	${\left|E^{*}\right|}_{min}$	●	×	×	×
	${\left|E^{*}\right|}_{max}$	×	●	×	×
	R	×	×	●	×
	C	×	×	×	●

●: influence, ×: no influence.

**Table 3:** Criteria for the quality of fit.

Criteria	$R_{*}^{2}$	$S_{e}/S_{y}$
Excellent	≥0.90	≤0.35
Good	0.70–0.89	0.36–0.55
Fair	0.40–0.69	0.56–0.75
Poor	0.20–0.39	0.76–0.89
Very Poor	≤0.19	≥0.90

**Table 4:** Average model parameters of master curves.

Mix. Type	Code	RAMBO Master Curve						Toranzos Master Curve				
		E_min_	E_max_	R	C_0_	B	C_1_	a	b	c	k	log(f_0_)
NB 50/70	AG	1 180	33 102	0.644	1.003	0.102	−0.286	0.155	−21.264	0.933	4427.572	3.242
PmB 25/55-65	BG	1 038	32 625	0.684	1.032	0.094	−0.298	0.139	−21.446	0.885	2904.633	3.296
RmB 45/80-55	CG	1 793	26 923	0.647	2.380	0.096	−0.273	0.466	2.645	0.693	11.116	3.355
RA 70/100 (B)	FG	286	35 674	0.742	1.905	0.071	−0.276	0.141	−22.341	1.409	3064.740	4.400
RA 70/100 (T)	GG	211	36 147	0.750	1.796	0.071	−0.283	0.090	−34.214	0.510	3468.389	3.744

**Table 5:** Comparison of fits of master curve models.

Mix. Type	Code	E* [MPa]			ϕ [°]		
		S_e_/S_y_	Adj. R^2^	R^2^	S_e_/S_y_	Adj. R^2^	R^2^
NB 50/70	AG1	0.1941	0.9507	0.9609	0.5024	0.6699	0.7585
	AG2	0.1495	0.9708	0.9775	0.2424	0.9232	0.9425
PmB 25/55-65	BG1	0.0815	0.9913	0.9930	0.2414	0.9238	0.9401
	BG2	0.1052	0.9855	0.9884	0.3668	0.8240	0.8663
RmB 45/80-55	CG1	0.0682	0.9939	0.9954	0.3592	0.8313	0.8660
	CG2	0.0859	0.9903	0.9932	0.3761	0.8150	0.8506
RA 70/100 (B)	FG1	0.1243	0.9825	0.9846	0.2781	0.9123	0.9237
	FG2	0.1279	0.9815	0.9834	0.3264	0.8792	0.8957
	FG3	0.2139	0.9481	0.9585	0.3212	0.8831	0.9027
RA 70/100 (T)	GG1	0.1927	0.9579	0.9664	0.3082	0.8923	0.9074
	GG2	0.2059	0.9520	0.9580	0.3672	0.8472	0.8684
	GG3	0.1122	0.9857	0.9875	0.3150	0.8876	0.9063
Colour bar of fitting quality: Excellent 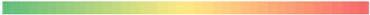 Very poor							

## Data Availability

Not applicable.
